# Inhibition of Melanization by a Parasitoid Serine Protease Homolog Venom Protein Requires Both the Clip and the Non-Catalytic Protease-Like Domains

**DOI:** 10.3390/insects2040509

**Published:** 2011-11-25

**Authors:** Pune Thomas, Sassan Asgari

**Affiliations:** School of Biological Sciences, The University of Queensland, St. Lucia QLD 4072, Australia; E-Mail: pune.thomas@uq.edu.au

**Keywords:** *Cotesia rubecula*, Vn50, prophenoloxidase: melanization, clip domin, serine protease homolog

## Abstract

Endoparasitoid wasps inject a variety of components into their host hemocoel at oviposition to facilitate successful development of their progeny. Among these are venom proteins which have been shown to play crucial roles in host regulation. A serine protease homolog (SPH)-like venom protein from *Cotesia rubecula* was previously shown to inhibit melanization in the host hemolymph by blocking activation of prophenoloxidase to phenoloxidase, a key enzyme in melanin formation. Similar to other SPHs, Vn50 consists of a clip and a protease-like (SPL) domain. Protein modeling demonstrated that Vn50 has a very similar structure to known SPHs and functional analysis of Vn50 domains expressed in insect cells indicated that neither of the domains on its own has an inhibitory effect on melanization.

## 1. Introduction

Melanization is a key component of the invertebrate immune system which is utilized to exclude and kill foreign bodies in the hemolymph [1,2]. The process of melanization is comprised of a strictly controlled cascade of protease enzymes. Prophenoloxidase (proPO) is an important enzyme in thiscascade which must first be catalysed by a proPO activating protease (PAP) to transform into its active form, phenoloxidase (PO) [3]. In addition, serine protease homologs (SPHs) of the clip-domain family that lack protease activity have been found to be essential for mediating activation of proPO by PAP [4]. Upon activation of SPHs, the protein is cleaved at the arginine or lysine residue which is located between the two cysteine residues forming the disulfide bond. Due to this disulfide bond, the two cleaved domains (clip and serine protease-like, SPL) remain attached [4]. Piao *et al.* (2005) presented the first crystal structure of a SPH, prophenoloxidase activating factor II (PPAF-II), from *Holotrichia diomphalia* (Coleoptera, Scarabaeidae), and demonstrated its main function in cleavage and activation of proPO [5]. Accordingly, the clip domain provides a module for binding proPO through the central cleft.

Endoparasitoids introduce a mixture of components during oviposition, including calyx fluid proteins, venom components, and symbiotic viruses and virus-like particles such as polydnaviruses [6]. These are utilized to inhibit the host immune system, or allow their progeny to evade it while they develop [7]. A 50 kDa SPH-like protein, Vn50, was isolated from the venom of *Cotesia rubecula* (Hymenoptera: Braconidae) which binds to proPAP, PAP, proPO and PO and inhibits activation of PO by competing with host SPHs for binding to proPO [8,9]. Unlike host SPHs that mediate activation of PO upon cleavage into the clip and SPL domains, Vn50 remains stably uncleaved for several days in the host hemolymph [9]. Cleavage of the hemolymph SPHs into the two domains are essential for mediating PO activation [4]. Vn50 is an example of the recruitment of an endogenous protein in the venom over the course of evolution, but with altered function [10].

**Figure 1:**
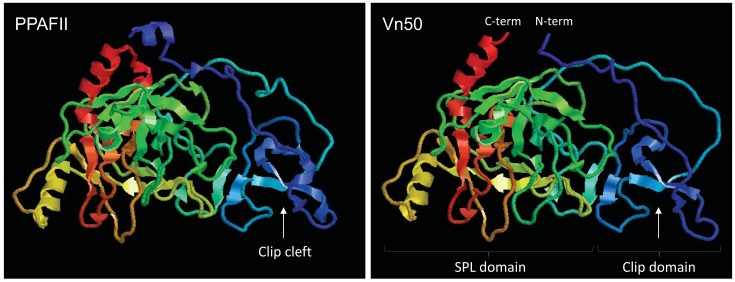
3D models constructed using ESyPred3D showing the overall predicted tertiary structure of Vn50 based on the crystal structure of serine protease homolog PPAFII from *Holotrichia diomphalia*. SPL, serine protease-like. C-terminus is shown in red and N-terminus is shown in dark blue.

To investigate if Vn50 has a similar structure to SPHs, tertiary structure predictions were generated using ESyPred3D v1.0 [11]. The model was generated using PPAF-II from *H. diomphalia* as a template [5]; a protein with 43% identity to Vn50 (E-value = 1e-82). The analysis showed that Vn50 is structurally analogous to PPAFs and forms a similar spatial configuration as PPAF-II (Figure 1). Based on the model, the clip domain in Vn50 contains loops and a central irregular β-sheet forming a clip cleft, like in PPAF-II, which is critical for binding of the protein to proPO. Similarly, the SPL domain of Vn50 had the overall structure of PPAF-II SPL domain with two clefts which may serve as docking sites for protein binding. Similar to other SPHs, in the SPL domain of Vn50 the active serine residue is replaced by a glycine [8]; therefore, Vn50 does not have enzyme activity.

**Figure 2:**
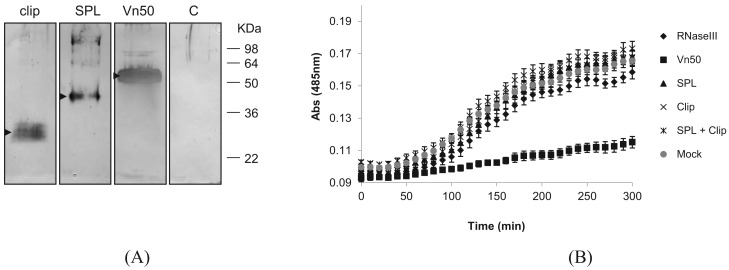
Expression and functional analysis of the clip domain, the serine protease-like (SPL) domain and the full-length Vn50. (**A**) Western blot analysis of media from Sf9 cells at 72 hours after infection with recombinant baculoviruses expressing the clip, SPL and the full-length Vn50. A polyclonal antibody against Vn50 was used. C, medium from cells infected with a control baculovirus expressing an ascovirus RNase III protein. Expressed proteins are indicated by arrows; (**B**) Melanization assays using the expressed proteins in (**A**) in combination with *P. rapae* hemolymph and l-DOPA as substrate. Mock, proteins purified from mock-infected Sf9 cells. Error bars indicate standard deviations of averages from six replicates.

To find out whether either of the Vn50 domains on their own have the PO inhibitory function, we expressed the two domains separately as well as the full-length Vn50, using the Bac-to-Bac baculovirus expression system in *Spodoptera frugiperda* Sf9 cells according to the manufacturer's instructions (Nitrogen). The corresponding coding regions for the two domains were first amplified with PCR by incorporating the restriction sites for direct cloning into the transfer vector pFastBac-HTa. For efficient secretion of the proteins, the coding sequences for the secretion signal of the honey bee, *Apis mellifera*, melittin gene (first 21 amino acids: MKFLVNVALVFMVVYISYIYA; GenBank accession no. CAA26038) were inserted into the transfer vector after which the corresponding Vn50 constructs were cloned. To confirm expression of the proteins, media were collected 72 h after infection and reduced in volume to approximately a tenth of their original volumes using a rotary evaporator and were run on a Western blot. The blot was then probed with an anti-Vn50 polyclonal antibody [8]. The proteins were all produced in fusion with 6× Histidine residues to ease purification. Complete Vn50, and the SPL and clip domains were all produced in infected cells and secreted into the medium generating bands of about 52 kDa, 42 kDa and 27 kDa respectively (Figure 2A). The expected sizes were 43 kDa, 32 kDa and 15 kDa, respectively. However, as previously shown [8], Vn50 is heavily glycosylated. Based on predictions, the clip domain has one N-glycosylation and six O-glycosylation putative sites and the protease-like domain with two N-glycosylation and one O-glycosylation putative sites. Medium from cells infected with a recombinant baculovirus expressing an ascovirus RNase III protein [12] used as control did not produce any band (Figure 2A).

Following confirmation of expression of the proteins, they were affinity purified from the medium collected from cells 72 h after infection using Ni-NTA agarose beads (BioRAD) that specifically bind to the 6× His-tag. These were then used for melanization assays. To provide hemolymph for the assays, 4^th^ instar *Pieris rapae* larvae were surface sterilized in 70% EtOH before bleeding from a proleg into 100 μL of ice-cold phosphate buffered saline (PBS). This was then centrifuged for 5 min at 800 × *g* and the supernatant (plasma) was transferred into a fresh tube. A 20 mM solution of L-3,4-dihydroxyphenylalanine (L-DOPA) in PBS was prepared immediately before use in order to avoid the effects of auto-oxidation. For each replicate, 10 μL of hemolymph was mixed with 10 μL of protein and 80 μL of L-DOPA. A control without hemolymph was also included to correct the data for auto-oxidation. Absorbance was measured at 485 nm each 10 min for 300 min.

Absorbance readings for the control (mock-infected) showed a significant increase in melanization compared to the full-length Vn50 which showed only slight melanization (Figure 2B; *P* < 0.0001 One-way ANOVA). The RNase III control, SPL domain only, clip domain only and a mixture of SPL and clip domains all showed increases in absorbance similar to the control (mock-infected) (Figure 2B; *P* = 2.773, One-way ANOVA). The results suggested that only the full-length Vn50 was successful in inhibiting melanization activity and that the expressed domains were not able to bind to proPO in an inhibitory fashion like Vn50, but also that they were unable to increase levels of melanization. Therefore, cleavage of Vn50 into the two domains is not all that is missing in Vn50 compared to other SPHs and the close interaction of the clip and protease domains is important in the activity of SPHs.

The activation of proPO to PO in the process of melanization is of major importance in the melanization process [3]. Usually, a non-enzymatic SPH that belongs to the clip-domain family of serine proteases is required to mediate this process which itself must first be activated [4]. The tightly interacting domains of the SPH, the SPL and clip domains must be cleaved in order for the protein to, in turn, promote the activation of proPO. However, the two domains remain attached by disulfide bonds. This was structurally confirmed by Piao *et al.* (2005) who published the crystal structure of PPAF-II, a clip-domain family SPH [5]. Vn50 3D modelling predicted a very similar structure to PPAF-II. To determine whether SPH-like activity in Vn50 was only lacking due to its inability to be cleaved in the host hemolymph, cleavage was simulated by expressing the domains separately using a baculovirus expression system. As expected, the whole Vn50 protein showed a strong inhibitory effect and levels of melanization were much lower than the controls. This confirmed the ability to express the functional venom protein in Sf9 cells, a much more natural form of expression than using bacterial cells. Addition of SPL domain alone had no effect on melanization, with levels being similar to that of the control. The same was seen for the clip domain and for a mixture containing both the SPL and clip domains. This is an interesting result as the lack of increase in melanization shows that a simulation of cleavage, with the two domains separated from one another, is not enough to give Vn50 a SPH-like activity in inducing melanization. The close interaction of the two domains after the cleavage must be important for activation of proPO. Furthermore, the domains, separately, no longer had an inhibitory effect on melanization as seen with the full-length Vn50. This suggests that the interaction between the two domains is important for the inhibitory binding of Vn50 to proPO.

## 2. Conclusions

Protein modeling showed that Vn50 has a very similar 3D structure to a known SPH (PPAF-II) with determined crystal structure, and functional analysis of Vn50 domains (clip and SPL) indicated that neither of the domains on its own has an inhibitory effect on melanization. These results give us deeper insight into two effects. Firstly, they reinforce the findings of past studies that emphasized the importance of the close interactions between the SPL and clip domains of SPHs and that this is essential for their activity. Secondly, they show the need for this interaction for successful binding to proPO and support the past assertion that Vn50's inhibitory activity is through competing with SPHs for the binding of proPO.
